# Asymptomatic giant coronary aneurysm in an adolescent with Behcet's syndrome

**DOI:** 10.1186/1546-0096-10-2

**Published:** 2012-01-06

**Authors:** Philip J Kahn, Yusuf Yazici, Michael Argilla, Monvadi Srichai, Deborah M Levy

**Affiliations:** 1 Department of Pediatrics, New York University Medical Center, 160 E. 32nd Street, New York, NY 10016, USA; 2 Department of Medicine, New York University Medical Center-Hospital for Joint Diseases, 246 e. 20th Street, New York, NY 10003, USA; 3 Department of Radiology and Medicine, New York University Medical Center, 560 First Avenue, New York, NY 10016, USA; 4 Department of Pediatrics, Hospital for Sick Children, 555 University Avenue, Toronto, ON, M5G 1X8, Canada

**Keywords:** Behcet's syndrome, vasculitis, coronary aneurysm

## Abstract

**Objective:**

Behcet's is an idiopathic multi-organ syndrome, which may have onset during childhood. Vascular involvement is uncommon, with rarely reported coronary aneurysm formation. We present a case report of a teenager girl who developed recalcitrant life-threatening Behcet's vasculitis, involving both small and large venous and arterial systems including a giant coronary aneurysm.

**Case report:**

De-identified data were collected retrospectively in case report format. Although our sixteen year old female with Behcet's vasculitis had resolution of many arterial aneurysms, she had persistent venous thrombosis of large vessels, as well as persistent, giant arterial aneurysms requiring intra-arterial coiling of a lumbar artery and coronary bypass grafting despite intensive immunosuppression including glucocorticoids, cyclophosphamide, infliximab, methotrexate, azathioprine and intravenous immunoglobulin.

**Conclusions:**

Vascular manifestations may be seen in Behcet's syndrome, including asymptomatic coronary aneurysm, which may be refractory to immunosuppression and ultimately require surgical intervention. Increased awareness is essential for prompt diagnosis and management.

## Background

Behcet's syndrome (BS) was initially described as a triad of symptoms including uveitis with oral and genital ulcerations [1]. It has since been expanded to include a constellation of recurring clinical manifestations with variable mucocutaneous, neurologic, ophthalmologic, cardiovascular, gastrointestinal, constitutional and musculoskeletal manifestations. Cardiovascular manifestations are uncommon; vascular lesions may affect both venous and arterial systems, and can occur in both large and small arteries. Aneurysms are a rare, potentially life-threatening manifestation of BS, with coronary aneurysm rarely reported.

We present the case of a teenage girl who developed medically refractory and life threatening Behcet's vasculitis, involving both small and large venous and arterial systems including a gigantic coronary aneurysm.

## Case report

The patient has given consent for the publication of her case report. A 16 year old female from the Dominican Republic presented with a three month history of malaise, fever, and anorexia and a ten pound weight loss. She also reported recurrent painful oral and genital ulcerations, as well as tender erythematous nodules over her lower legs, suggestive of erythema nodosum. Her laboratory evaluation was significant for normocytic anemia and elevated acute phase reactants.

As part of her evaluation for fever, a chest CT and subsequent magnetic resonance imaging (MRI), including MR angiography and venography revealed multiple arterial aneurysms of bilateral renal and splenic vessels (the largest splenic aneurysm measured 2.8 × 1.9 cm), as well as multiple intercostal and lumbar arterial aneurysms (the largest measured 2.9 × 2.5 cm at the level of the left psoas muscle). There was extensive venous thrombosis of the right jugular vein, inominate vein and within the upper segment of the superior vena cava (SVC), although patency was observed below the level of the enlarged azygous vein, with multiple prominent intercostal collaterals within the left hemithorax.

Intensive immunosuppression was initiated for the presumptive diagnosis of BS, including daily oral cyclophosphamide (2 mg/kg/day) and three daily infusions of intravenous pulse solumedrol (1,000 mg) followed by 60 mg of prednisone daily; the latter was tapered slowly over the next six months. Therapeutic anticoagulation was initiated with low molecular weight heparin. Small patches of cutaneous induration were observed 24-48 hours after subcutaneous injection, suggestive of a pathergy-like reaction, although without classic papule or pustule formation.

Follow-up MRI at four months demonstrated complete resolution of splenic, renal, intercostal and most lumbar aneurysms; however the largest lumbar artery aneurysm did not regress. Fever and oral ulcerations resolved and she regained the lost weight. The elevated acute phase reactants also normalized. After six months cyclophosphamide was discontinued and maintenance therapy with monthly infliximab infusions (10 mg/kg/dose) and weekly oral methotrexate (25 mg) were initiated. One year after presentation MRI demonstrated enlargement of the asymptomatic lumbar artery aneurysm, which now measured 5.7 × 5.0 × 5.7 cm. With no new arterial aneurysms and normal inflammatory markers, there were no overt signs of active disease. She underwent successful intravascular coiling of the lumbar artery aneurysm.

At 18 month follow-up, MRI revealed persistent venous thrombosis of the right jugular vein and venal caval system, without new thrombi. Anticoagulation was discontinued and low dose daily aspirin (81 mg) was initiated. Although there were no clinical, imaging or laboratory data suggestive of active vasculitis, the patient's immunosuppression was modified to receive three additional cycles of monthly intravenous cyclophosphamide (increasing doses of 500, 750 and 1,000 mg/m2) given the possibility that venous clot signified ongoing inflammation.

**Figure 1:**
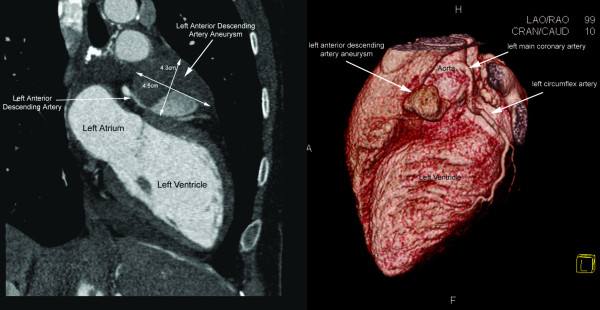
**Cardiac computed tomography images in 2D oblique sagittal view (left) and 3D volume reconstructed view (right) demonstrating partially thrombosed coronary artery aneurysm involving the proximal segment of the left anterior descending artery**. The aneurysm cavity (double-headed arrows) demonstrates partial thrombosis (low attenuation or darker areas) and widely patent left anterior descending artery lumen (high attenuation or brighter areas) on the 2D images (left).

At two years, MRI demonstrated an asymptomatic giant (4.5 × 4.3 × 3.8 cm) predominantly thrombosed and partially calcified saccular aneurysm of the proximal left anterior descending (LAD) coronary artery. This was confirmed by coronary CT angiography (Figure 1). Left ventricle size and function were normal, with no segmental wall motion abnormalities. Cardiac stress testing did not reveal any symptomatic or electrocardiographic findings of myocardial ischemia during exercise.

Despite escalation of therapy with daily oral cyclophosphamide (2.3 mg/kg/day), high dose steroids and warfarin, five months later an MRA revealed enlargement (6.7 × 4.2 × 4.9 cm) of the LAD aneurysm. Immunosuppression was switched to monthly infliximab infusions (10 mg/kg), daily azathioprine 100 mg and three monthly infusions of intravenous immunoglobulin (IVIG, 2 gm/kg). Four months later, echocardiography demonstrated further asymptomatic enlargement (8 cm × 6 cm) of the LAD aneurysm. She continued to have no clinical or laboratory signs of active BS, and successfully underwent two-vessel coronary artery bypass and was placed on daily prednisone (40 mg) and azathioprine (150 mg) postoperatively. At five month follow-up she continues to have no signs or symptoms of active BS or coronary disease on continued azathioprine therapy.

## Discussion

Expanding on the initial description of BS by a dermatologist who noted the triad of uveitis with oral and genital ulcerations in three patients, the International Study Group Classification criteria (Table 1) define BS as an idiopathic multi-system vasculitis excluding other disease entities [1,2]. Although originally described in patients along the "Silk Road", the syndrome occurs worldwide with a prevalence of 20-421 per 100,000 adults, though exact numbers are uncertain [3].

**Table 1:** International Study Group diagnostic (classification) criteria for Behcet's syndrome (1990)[2]

Recurrent oral ulceration	Minor aphthous, major aphthous, or herpetiform ulceration observed by physician or patient recurring at least three times in one 12 month period
Plus two of the following	
Recurrent genital ulceration	Aphthous ulceration or scarring, observed by physician or patient
Eye lesions	Anterior uveitis, posterior uveitis, cells in the vitreous on slit-lamp examination; or retinal vasculitis observed by an ophthalmologist
Skin lesions	Erythema nodosum observed by the physician or patient, pseudofolliculitis, papulopustular lesions or acneiform nodules observed by physician in post adolescent patients not on corticosteroid treatment
Pathergy	Development of a papule or pustule 24-48 hours after intradermal injection of the skin with a 20 gauge needle.
Findings applicable only in the absence of other clinical explanations	

The typical age of onset is in the third decade of life; however up to 20% of patients have onset before age 16 [4]. Our patient represents an atypical example of childhood-onset BS, with extensive venous thrombosis and arterial aneurysms, and progressive vascular disease despite aggressive immunosuppressive therapy, ultimately requiring endovascular coiling and coronary bypass surgery. Vascular involvement is reported in up to 28% of patients [5,6]. Venous manifestations occur in 10-40%, including superficial thrombophlebitis as well as thrombosis of large veins such as the cerebral venous sinuses, vena cavae, and jugular venous systems, often with extensive, reactive collateralization [7]. Arterial lesions are less common, occurring in up to 7% of patients, and manifest as aneurysm, thrombosis or stenosis, with the most commonly affected arteries including the abdominal aorta, femoral arteries and pulmonary arteries [5,8-10]. Aneurysm is more frequent than arterial occlusion, although both may occur simultaneously [8,9,11]. Coronary aneurysm formation is rare, and the outcome ranges from resolution, persistent morphology, stenosis, occlusion, dissection or rupture [12]. Although used, the clinical utility of radiographic evaluation by MRI or angiography, and laboratory markers is undetermined.

There are few reports of the histology of Behcet's vascular lesions which demonstrate non-specific aortitis, with elements of active inflammation, scar or both [5]. In contrast to the vasculitic inflammatory phase, the scar stage consists of fibrous thickening of the intima and adventitia. It is likely that the true incidence of vascular lesions is underestimated, perhaps related to the fact that patients may have significant vascular lesions despite being asymptomatic. In fact, a study of 170 autopsies of patients with BS, arterial involvement was discovered in 57 patients [13].

The development of a coronary aneurysm as well as its increasing size despite treatment is concerning, although it is possible that this may have been the inevitable consequence of the arteritis, with aneurysmal emergence as the result of degradation of the extracellular matrix of the coronary artery. Given the rarity and heterogeneity of the disease and lack of controlled trials the optimal treatment of patients with BS has not been standardized. Traditional therapies have included glucocorticoids and colchicine; however more recently tumor necrosis factor blocking agents, such as infliximab, have also demonstrated clinical utility [14]. Based on an extensive literature review by the European League Against Rheumatism (EULAR), the use of cyclophosphamide is reserved for severe vascular involvement, based largely on retrospective studies as no randomized controlled trial has been performed [15].

In childhood, aneurysm of the coronary arteries are most commonly observed in Kawasaki's disease, a childhood vasculitis with coronary aneurysm formation in up to 20% of untreated children [16]. The risk of aneurysm with IVIG treatment is reduced to 5%. Although it is unknown in KD whether IVIG alters the natural course of the aneurysm after its formation, in this case IVIG seemed a reasonable strategy in this recalcitrant patient, although data pertaining to its use in Behcet's vasculitis are absent.

The decision and timing of intravascular or open surgery in Behcet's aneurysm is controversial. A surgical procedure may be required in the setting of organ ischemia, acute rupture, or increasing size. Surgery may be complicated by occlusion or recurrence of the aneurysm, due to a pathergy-like vascular reaction to surgical trauma [8]. Surmising that the likelihood of a postoperative pathergy-like response is more likely during the inflammatory stage of disease, it is beneficial to have reassuring evidence that there is not ongoing disease. Though recognizing the unreliable validity of normal inflammatory markers and imaging studies, even the asymptomatic patient should be studied before surgery is pursued. The use of postoperative glucocorticoids has been demonstrated to minimize the risk of recurrence of aneurysms, more often given in combination with other immunosuppressants, such as azathioprine [10].

Although uncommonly seen, coronary aneurysm may result in significant morbidity and mortality in patients with BS. The efficacy of aggressive immunosuppression is unclear, as many patients may be refractory to such therapy and ultimately require a surgical procedure unless succumbing to their disease before an appropriate surgical intervention is offered. Increased awareness regarding potentially asymptomatic vascular complications in is critical in the timely care of the patient with BS.

## Consent

Written informed consent was obtained from the patient for publication of this Case Report and any accompanying images. A copy of the written consent is available for review by the Editor-in-Chief of this journal.

## Competing interests

The authors declare that they have no competing interests.

## Authors' contributions

PK made substantial contributions to the manuscript in the following ways: 1) conception and design, acquisition and interpretation of data; 2) drafting the manuscript and revising it critically for important intellectual content; 3) has given final approval of the version to be published. YY made substantial contributions to the manuscript in the following ways: 1) conception and design, acquisition and interpretation of data; 2) drafting the manuscript and revising it critically for important intellectual content; 3) has given final approval of the version to be published. MM made substantial contributions to the manuscript in the following ways: 1) conception and design, acquisition and interpretation of data; 2) drafting the manuscript and revising it critically for important intellectual content; 3) has given final approval of the version to be published. MS made substantial contributions to the manuscript in the following ways: 1) conception and design, acquisition and interpretation of data; 2) drafting the manuscript and revising it critically for important intellectual content; 3) has given final approval of the version to be published. DL made substantial contributions to the manuscript in the following ways: 1) conception and design, acquisition and interpretation of data; 2) drafting the manuscript and revising it critically for important intellectual content; 3) has given final approval of the version to be published.
